# Nomogram based on spleen volume expansion rate predicts esophagogastric varices bleeding risk in patients with hepatitis B liver cirrhosis

**DOI:** 10.3389/fsurg.2022.1019952

**Published:** 2022-11-16

**Authors:** Jianghong Li, Junjie Li, Qian Ji, Zhenglu Wang, Honghai Wang, Sai Zhang, Shunli Fan, Hao Wang, Dejun Kong, Jiashu Ren, Yunhui Zhou, Ruining Yang, Hong Zheng

**Affiliations:** ^1^ First Central Clinical College, Tianjin Medical University, Tianjin, China; ^2^ Department of Organ Transplantation, Tianjin First Central Hospital, Tianjin, China; ^3^ Department of Radiology, Tianjin First Central Hospital, Tianjin, China; ^4^ Key Laboratory of Transplant Medicine, Chinese Academy of Medical Sciences, First Central Clinical College, Tianjin Medical University, Tianjin, China; ^5^ School of Medicine, Nankai University, Tianjin, China; ^6^ Tianjin Key Laboratory for Organ Transplantation, Tianjin First Central Hospital, First Central Clinical College, Tianjin Medical University, Tianjin, China

**Keywords:** liver and spleen volume, serum sodium, esophagogastric varices bleeding, hepatitis B cirrhosis, nomogram

## Abstract

**Background:**

We aimed to explore the risk factors for hemorrhage of esophagogastric varices (EGVs) in patients with hepatitis B cirrhosis and to construct a novel nomogram model based on the spleen volume expansion rate to predict the risk of esophagogastric varices bleeding.

**Methods:**

Univariate and multivariate logistic regression analysis was used to analyze the risk factors for EGVs bleeding. Nomograms were established based on the multivariate analysis results. The predictive accuracy of the nomograms was assessed using the area under the curve (AUC or C-index) of the receiver operating characteristic (ROC) and calibration curves. Decision curve analysis was used to determine the clinical benefit of the nomogram. We created a nomogram of the best predictive models.

**Results:**

A total of 142 patients' hepatitis B cirrhosis with esophagogastric varices were included in this study, of whom 85 (59.9%) had a history of EGVs bleeding and 57 (40.1%) had no EGVs bleeding. The spleen volume expansion rate, serum sodium levels (mmol/L), hemoglobin levels (g/L), and prothrombin time (s) were independent predictors for EGVs bleeding in patients with hepatitis B liver cirrhosis (*P* < 0.05). The above predictors were included in the nomogram prediction model. The area under the ROC curve (AUROC) of the nomogram was 0.781, the C-index obtained by internal validation was 0.757, and the calibration prediction curve fit well with the ideal curve. The AUROCs of the PLT-MELD and APRI were 0.648 and 0.548, respectively.

**Conclusion:**

In this study, a novel nomogram for predicting the risk of EGVs bleeding in patients with hepatitis B cirrhosis was successfully constructed by combining the spleen volume expansion rate, serum sodium levels, hemoglobin levels, and prothrombin time. The predictive model can provide clinicians with a reference to help them make clinical decisions.

## Background

Hepatitis B virus (HBV) infection is the leading cause of chronic liver disease. More than 240 million people worldwide are chronically infected with HBV, and approximately 15%–40% of untreated chronically infected individuals will develop cirrhosis (1, 2). With the development of liver cirrhosis, many related clinical complications will occur, among which splenomegaly and esophagogastric varices (EGVs) are most common; EGVs are also the main cause of death (3). Although effective treatment of EGVs has improved in recent decades, the mortality rate of variceal bleeding remains as high as at least 20% within 6 weeks (4–6). If the EGVs rupture hemorrhage can be correctly predicted before EGVs hemorrhage attack, the EGVs bleeding related mortality and related complications can be effectively reduced. Therefore, it is particularly important to screen EGVs in patients with hepatitis B cirrhosis (HBC) in advance and prevent bleeding. The spleen in cirrhotic patients with portal hypertension can be palpated on physical examination and the volume of the spleen as measured by CT is increased, as has been demonstrated in several studies (7–9). Previous studies have shown that portal vein diameter and liver/spleen volume ratio measured by computed tomography (CT) can predict the presence of portal hypertension and EGVs (10–12). Spleen volume, right liver volume, and liver volume correlated with the presence of EGVs (13–15). So, we choose CT examination to evaluate liver and spleen volume and complications related to liver cirrhosis in patients with liver cirrhosis. At present, there is no clear definition for the description of spleen volume expansion rate in patients with liver cirrhosis, we defined the spleen volume expansion rate as the ratio of the difference between the actual spleen volume measured by CT and the standard spleen volume to the standard spleen volume. Many studies have constructed noninvasive models for predicting the occurrence of bleeding in EGVs, but there is no report on the nomogram prediction model based on CT and other imaging techniques to evaluate the combination of the spleen volume expansion rate (SVER) and clinical laboratory test results. In this study, we aimed to construct an innovative nomogram model based on the computed splenic volume expansion rate calculated by CT and the results of laboratory examinations, hoping to help clinicians predict the risk of EGVs bleeding and take intervention measures in advance to prevent adverse clinical events.

## Methods

### Patients

The data were collected from patients admitted to our hospital from December 2015 to January 2021 and diagnosed with hepatitis B cirrhosis with esophagogastric varices. The results of the first laboratory test and imaging examination after admission were retrospectively collected. The enrolled patients were divided into two groups according to whether esophagogastric varices bleeding occurred, namely, Bleeding group and Nonbleeding group. The results of the first laboratory examination, imaging examination and other clinical symptoms in the two groups were analyzed. Inclusion criteria were as follows: (1) age ≥18 years; (2) patients diagnosed with HBC; and (3) complete imaging data. Exclusion criteria were as follows: (1) patients with other types of liver cirrhosis, such as alcoholic liver cirrhosis, other hepatitis virus-related liver cirrhosis, and autoimmune liver cirrhosis; (2) a history of hepatectomy and/or splenectomy; (3) splenic cysts (>1 cm in diameter); (4) patients with hematological diseases; (5) patients with other diseases that affect spleen volume; and (6) patients with severe cardiovascular and cerebrovascular diseases, metabolic diseases, and/or renal incompetence.

### Image parameter measurement

All patients underwent enhanced CT and ultrasonography examination. The upper abdominal enhanced CT examination was performed using a Siemens Definition dual source CT scanner (Siemens, Germany). Liver ultrasonography examination was performed using a Siemens S2000 ultrasound (OXANA3, Germany).

**Figure 1:**
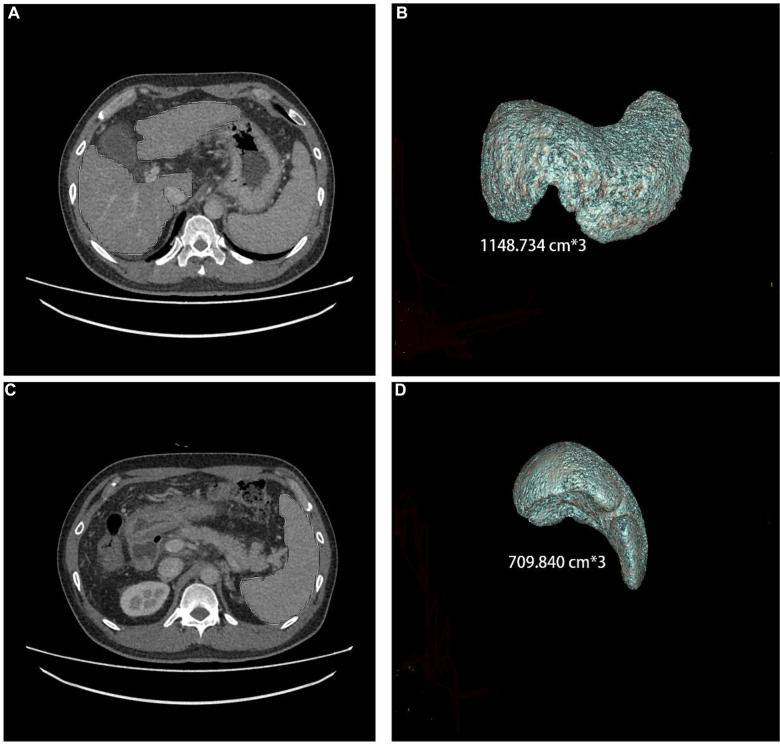
Liver and spleen volume, as measured by CT, in a 44-year-old male who has had hepatitis B cirrhosis for 20 years.

The CT and ultrasonography data from the patients were retrospectively collected. The patients' image data included CTLV, CTSV, PVD, PVV, and PVT. The patients' upper abdominal CT thin slice images were selected in the portal venous phase. The actual liver and spleen volumes were measured using an IQQA-Liver workstation (EDDA Company, USA) (Figure 1). The results were reviewed by two radiologists (with 13 years and 5 years of experience with abdominal CT imaging). During the whole measurement process, large blood vessels, the gallbladder, the falciform ligament of the liver, the ribs, the pancreas, the gastrointestinal tract, the abdominal wall, and other tissues were avoided to minimize human error and improve the accuracy of measurements.

### Clinical laboratory data and observations

The first peripheral venous blood was drawn from each patient in the early morning on an empty stomach. The following data were recorded: red blood cell count (10^12^/L), platelet count (PLT) (10^9^/L), hemoglobin (Hb) levels (g/L), prothrombin time (PT) (s), total bilirubin (TBil) levels (μmol/L), aspartate aminotransferase (AST) levels (U/L), alanine aminotransferase (ALT) levels (U/L), serum sodium (SNA) levels (mmol/L), daily alcohol consumption (g/d), height (m), weight (kg), body surface area (BSA), the Child–Pugh grade, the Child–Pugh score, and the Model for End-stage Liver Disease (MELD) score. Serum biochemical parameters were measured using a Cobas c701 automatic biochemical analysis system.

### Formulas

BSA was calculated according to the Mosteller formula: BSA = √[Wt (kg) × Ht (cm)/3600] (16). The MELD score was calculated as follows: MELD score = 9.57 × LN (creatinine) + 3.78 × LN (bilirubin) + 11.2 × LN (INR) + 6.43, where creatinine (mg/dl), bilirubin (mg/dl), and INR values below 1.0 were set to 1 (17, 18). The corresponding standard liver volume (SLV) and standard spleen volume (SSV) were calculated according to the patient's height (cm), weight (kg), and BSA as follows: SLV (cm^3^) = 858.186 × BSA − 393.349 (*R*^2^ = 0.350) and SSV (cm^3^) = 188.813 × BSA − 140.981 (*R*^2^ = 0.126). Other parameters included the following: the ratio of liver volume change rate [LVCR = CT-measured liver volume (CTLV) − SLV] to SLV, where LVCR < 0 means the liver has shrunk; the ratio of spleen volume expansion (SVER) = [CT-measured spleen volume (CTSV) − SSV] to SSV; the ratio of CT-measured liver volume to CTSV (CTLSVR); and the AST to PLT ratio (APRI) (8, 19).

### Statistical analysis

Statistical analysis was performed using IBM SPSS Statistics version 26.0 (SPSS Inc., Chicago, United States) and RStudio 4.1.2 (http://www.R-project.org). For comparisons between the two groups, continuous variables that follow a normal distribution were analyzed using the t-test and are expressed as mean ± standard deviation; continuous variables that follow a nonnormal distribution were analyzed using the Mann–Whitney U test and are expressed as the median [interquartile range (IQR)]; and categorical variables were analyzed using the chi-square (*χ*^2^) test and the McNemar test and are expressed as numbers (percentages). Univariate logistic regression analysis was conducted to predict the risk factors associated with bleeding from EGVs in patients with HBC. After a diagnosis of collinearity for variables with *P* < 0.05 in univariate logistic regression analysis, multivariate logistic regression analysis was performed for variables with *P* < 0.05 and VIF < 3.0 to identify risk factors that independently affected the outcome. On the basis of the multivariate logistic regression analysis results (*P* < 0.05), a nomogram model for predicting the bleeding risk of EGVs was constructed using the R package rms in RStudio 4.1.2, and the corresponding receiver operating characteristic (ROC) curve, calibration curve and clinical decision curve. Internal validation was performed using the bootstrap method (1,000 iterations) to verify the accuracy of the prediction model and the consistency of the calibration curve assessment. Finally, the clinical benefit of the model was evaluated. Model discrimination was assessed by calculating the area under the ROC curve (AUROC or C-index). Hosmer–Lemeshow goodness-of-fit test was used to evaluate whether the predicted probability and actual probability calculated by the model fit well. *P* > 0.05 was an acceptable level at which the model's estimate fit the data, indicating that the model worked well. Finally, the integrated discrimination improvement (IDI) was used to evaluate the overall improvement of the model. A clinical decision curve can describe the net benefit given by a nomogram and can help determine whether the benefits of making clinical decisions based on this model outweigh the risks. A two-sided *P* < 0.05 was considered statistically significant. The interclass correlation coefficient (ICC) was used to assess the interobserver reliability of measurements. ICC < 0.40, 0.40–0.75, >0.75 indicated poor, fair to good, and excellent agreement, respectively.

## Results

### Baseline dates and clinical characteristics

According to the inclusion and exclusion criteria, we collected data of 142 patients’ hepatitis B cirrhosis with esophagogastric varices. Their mean age was 50.38 ± 9.91 years; 126 (88.7%) were male and 16 were (11.3%) female; 38 (26.8%) patients had portal vein thrombosis and 104 (73.2%) had no portal vein thrombosis; and 85 (59.9%) patients had an EGVs bleeding history and 57 (40.1%) had no bleeding history of EGVs (Table 1). The results were reviewed by two radiologists: CT analyses observer repeatability in the analyses of had excellent interobserver agreement with ICCs of 0.946 (95%CI: 0.913–0.967) and 0.904 (95%CI: 0.846–0.941).

**Table 1:** Baseline features of study population.

Parameter		Bleeding group (85)	Nonbleeding group (57)	Total (142)	*P* value
Age (y)		49.7 ± 9.6	51.4 ± 10.4	50.4 ± 9.9	0.299
Sex (%)	M	77 (90.6%)	49 (86.0%)	126 (88.7%)	0.393
	F	8 (9.4%)	8 (14.0%)	16 (11.3%)	
PVT (%)	None	60 (70.6%)	44 (77.2%)	104 (73.2%)	0.498
	Exist	25 (29.4%)	13 (22.8%)	38 (26.8%)	
Child class (%)	Class A	22 (25.9%)	4 (7.0%)	26 (18.3%)	0.017
	Class B	36 (42.4%)	31 (54.4%)	67 (47.2%)	
	Class C	27 (31.8%)	22 (38.6%)	49 (34.5%)	
Ascites (%)	None	27 (31.8%)	15 (26.3%)	42 (29.6%)	0.091
	Exist	58 (68.2%)	42 (73.7%)	100 (70.4%)	
Disease period (year)		18 (10,20)	20 (10,28)	20 (10,21)	0.122
Height (m)		1.71 ± 0.06	1.72 ± 0.06	1.71 ± 0.06	0.623
Weight (kg)		66.50 (61.00, 74.65)	70.00 (63.25, 81.00)	69.50 (62.00, 77.00)	0.085
SLV (cm^3^)		1169.446 ± 155.051	1186.916 ± 134.226	1176.459 ± 146.805	0.489
SSV (cm^3^)		202.856 ± 34.113	206.700 ± 29.532	204.399 ± 32.399	0.489
CTLV (cm^3^)		1006.667 (839.846, 1211.671)	1049.199 (866.616, 1200.813)	1035.799 (851.524,1203.977)	0.619
CTSV (cm^3^)		1166.327 (722.389, 1588.644)	739.784 (515.766, 1139.746)	997.456 (648.684,1433.018)	<0.001
LVCR		−0.119 (−0.283, 0.060)	−0.126 (−0.280, 0.046)	−0.123 (−0.281,0.054)	0.879
SVER		4.330 (2.811, 7.197)	2.686 (1.492, 4.579)	3.969 (2.186,5.917)	<0.001
CTLSVR		0.907 (0.657, 1.265)	1.282 (0.877, 2.104)	1.051 (0.697,1.530)	<0.001
PVV (cm/s)		14.4 (10.4, 18.4)	14.2 (10.0, 18.65)	14.3 (10.0,18.6)	0.965
PVD (cm)		1.2 (1.0, 1.5)	1.1 (1.0, 1.4)	1.20 (1.0,1.5)	0.109
RBC (10^12^/L)		3.35 ± 0.67	3.48 ± 0.72	3.40 ± 0.69	0.257
Hb (g/L)		95.29 ± 21.53	110.79 ± 24.00	101.51 ± 23.73	<0.001
ALT (U/L)		20.8 (14.3,32.7)	32.4 (20.4,51.9)	24.7 (16.5,38.8)	<0.001
AST (U/L)		28.3 (21.2,46.3)	29.2 (16.2,50.2)	28.8 (20.6,47.9)	0.492
TBil (μmol/L)		26.0 (18.6,55.2)	43.5 (24.2,94.8)	32.4 (19.2,63.7)	0.006
SNA (mmol/L)		139.8 (138.0,142.0)	137.8 (134.3,140.0)	139.3 (136.3,141.5)	0.001
PT (s)		16.9 (14.4,20.1)	18.6 (15.9,27.9)	17.50 (14.90,20.95)	0.005
PLT (10^9^/L)		50.0 (37.0,73.5)	60.0 (44.0,86.0)	55.0 (40.0,80.0)	0.066
Child score		8.0 (7.0,10.0)	9.0 (8.0,11.0)	9.0 (7.0,11.0)	0.016
MELD score		14.0 (10.0,17.0)	15.0 (12.0,21.0)	14.0 (10.0,18.0)	0.026
Alcohol (g/d)		0.0 (0.0,100.0)	0.0 (0.0,25.0)	0.0 (0.0,62.5)	0.680

SLV, standard liver volume; SSV, standard spleen volume; CTLV, CT measured liver volume; CTSV, CT measured spleen volume; CTLSVR, CT measured liver volume to CT measured spleen volume ratio; PVD, portal vein diameter; PVV, portal vein velocity; PVT, portal vein thrombosis.

### Identification of risk factors for esophagogastric varices bleeding with hepatitis B cirrhosis

Univariate logistic regression analysis showed that the CTSV (OR = 1.001, 95%CI: 1.000–1.002, *P* = 0.003), SVER (OR = 1.248, 95%CI: 1.083–1.439, *P* = 0.002), CTLSVR (OR = 0.520, 95%CI: 0.341–0.793, *P* = 0.002), TBil (OR = 0.996, 95%CI: 0.991–1.000, *P* = 0.08), SNA (OR = 1.132, 95%CI: 1.042–1.230, *P* = 0.003), PT (OR = 0.915, 95%CI: 0.864–0.970, *P* = 0.003), Hb (OR = 0.970, 95%CI: 0.955–0.986, *P* < 0.001), Child–Pugh score (OR = 0.834, 95%CI: 0.719–0.968, *P* = 0.015), and MELD score (OR = 0.930, 95%CI: 0.877–0.986, *P* = 0.015) were significantly different between the two groups (*P* < 0.05); PVT (OR = 1.410, 95%CI: 0.650–3.061, *P* = 0.385), ascites (OR = 0.767, 95%CI: 0.364–1.617, *P* = 0.486), CTLV (OR = 1.000, 95%CI: 0.999–1.001, *P* = 0.997), LVCR (OR = 1.233, 95%CI: 0.329–4.619, *P* = 0.756) were not significantly different between the two groups (*P* > 0.05). Multivariate logistic regression analysis showed that SVER [*P* = 0.020, odds ratio (OR) = 1.195, 95%CI: 1.028–1.388], SNA (*P* = 0.025, OR = 1.119, 95% CI: 1.014–1.235), PT (*P* = 0.047, OR = 0.933, 95% CI: 0.872–0.999), and Hb (*P* = 0.006, OR = 0.975, 95% CI: 0.958–0.993) were the independent risk factors for bleeding from EGVs (Table 2). Considering that there may be differences between different genders, we performed the corresponding logistic regression analysis, and the results showed that there was no statistical difference in spleen volume between different genders (aOR = 1.001, 95%CI 0.999–1.003, *P* = 0.151).

**Table 2:** Univariate and multivariate logistic regression analysis of bleeding in EGEs.

Variable	Univariate analysis		*P* value	Multivariate analysis		*P* value
	OR	95%CI		OR	95%CI	
PVT^a^	1.410	0.650–3.061	0.385			
Ascites^b^	0.767	0.364–1.617	0.486			
CTLV	1.000	0.999–1.001	0.977			
LVCR	1.233	0.329–4.619	0.756			
CTSV	1.001	1.000–1.002	0.003			
SVER	1.248	1.083–1.439	0.002	1.195	1.028–1.388	0.02
CTLSVR	0.52	0.341–0.793	0.002			
SNA	1.132	1.042–1.230	0.003	1.119	1.014–1.235	0.025
TBil	0.996	0.991–1.000	0.08			
PT	0.915	0.864–0.970	0.003	0.933	0.872–0.999	0.047
Child score	0.834	0.719–0.968	0.015			
Hb	0.97	0.955–0.986	<0.001	0.975	0.958–0.993	0.006
MELD score	0.93	0.877–0.986	0.015			

^a^ The absence of portal vein embolism was used as the reference category.

^b^ No ascites was used as the reference category.

### Construct a nomogram to predict the risk of EGVs bleeding

**Figure 2:**
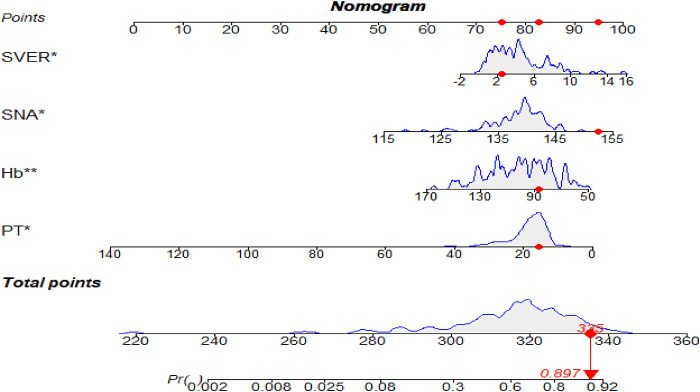
The nomogram for predicting the bleeding risk of esophagogastric varices in patients with hepatitis B cirrhosis. A 59-year-old male patient with hepatitis B cirrhosis for 20 years. The total score and the density map of SVER, SNA, Hb, and PT are shown. The importance of each variable is ordered according to the standard deviation on the nomogram scale. To use the nomogram, individual patient-specific points (red dots) are located on each variable axis. Mark the red dots on the points axis to determine the number of points each variable received; the sum of these points (335) lies on the total points axis. Draw a line down to the probability axis to identify the probability of esophagogastric variceal bleeding (89.7%).

**Figure 3:**
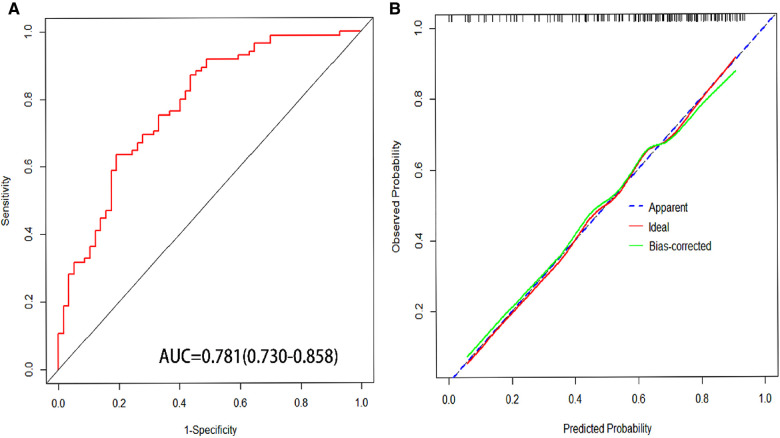
(**A**) Area under receiver operating characteristic (AUROC) of nomogram for predicting esophagogastric variceal bleeding with hepatitis B cirrhosis. (**B**) The calibration curves for predicting esophagogastric variceal bleeding with hepatitis B cirrhosis.

The predictive nomogram was constructed according to the multivariate logistic regression analysis results (*P* < 0.05). The independent risk factors included SVER, SNA, PT, and Hb. On the nomogram, the value on the scale line of each predictor corresponds to the points on the scale, and the sum of all the index scores is the total points; the total score corresponds to the predicted value of the risk of EGVs bleeding (Figure 2). The area under the ROC curve (AUROC) of the nomogram was 0.781 (95% CI: 0.703–0.858) and the cutoff value was 0.743 (sensitivity 80.7% and specificity 63.5%) (Figure 3A). The Hosmer-Lemeshow goodness-of-fit test value was *R*^2^ = 5.675, *P* = 0.684. The calibration plot of the model showed that the calibration prediction curve fit well with the ideal curve, suggesting that the model has a good predictive value for the occurrence of EGVs bleeding (Figure 3B). We compared the prediction model constructed in this study with the PLT-MELD model, and the results showed that our prediction model improved 19.7% over the PLT-MELD model [IDI (95% CI) = 0.197 (0.123–0. 270), *P* < 0.001], and improved 23.7% over the APRI model [IDI (95% CI) = 0.237 (0.158–0. 315), *P* < 0.001].

### Comparison of prediction accuracy of the nomogram and clinical decision curve analysis

**Figure 4:**
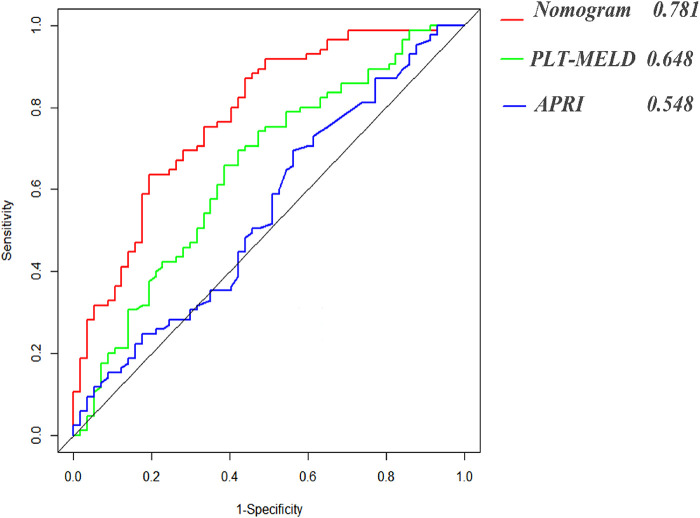
Area under receiver operating characteristic (ROC) comparison of nomogram, PLT-MELD and APRI.

The constructed nomograms indicated better accuracy compared to the APRI and PLT-MELD (20) in predicting EGVs bleeding in patients with HBC (Figure 4). The constructed nomogram had the higher AUROC values (0.781, sensitivity 80.7% and specificity 63.5%) than the PLT-MELD (0.648, sensitivity 69.4% and specificity57.9%) and the APRI (0.548, sensitivity 70.6% and specificity 43.9%) (Table 3). These results indicate that the constructed nomogram can be helpful in predicting EGVs bleeding in patients' hepatitis B cirrhosis with esophagogastric varices.

**Table 3:** Predictive performances of nomograms, PLT-MELD and APRI for esophagogastric variceal bleeding with hepatitis B cirrhosis.

Parameter	AUROC (95% CI)	*P*	Cutoff	Se (%)	Sp (%)
Nomogram	0.781 (0.703–0.858)	<0.001	0.743	80.7	63.5
PLT-MELD	0.648 (0.554–0.742)	0.003	0.444	69.4	57.9
APRI	0.548 (0.450–0.646)	0.333	0.451	70.6	43.9

AUROC, area under ROC; Se, sensitivity; Sp, specificity.

**Figure 5:**
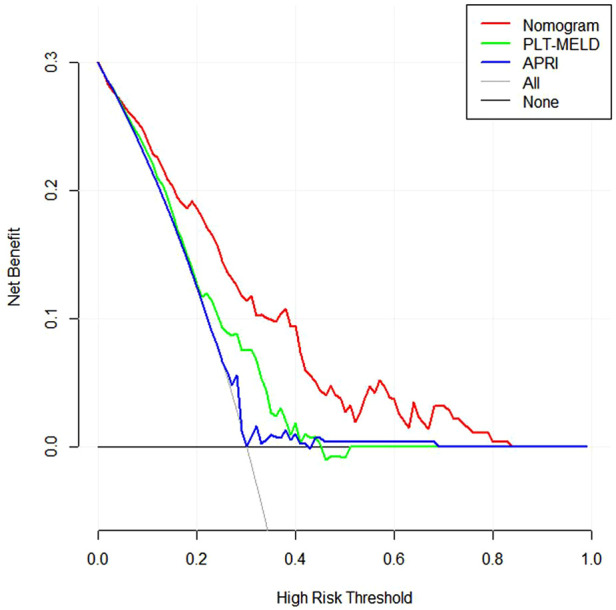
Decision curves for prediction of the net benefit of the constructed nomogram, PLT-MELD and APRI. Gray line: net benefit of a strategy of treating all patients with esophagogastric varices in hepatitis B cirrhosis. Horizontal black line: net benefit of treating no patients with esophagogastric varices in hepatitis B cirrhosis. Colored lines: net benefit of a strategy of treating patients according to nomogram, PLT-MELD and APRI. Red line: screen based on the nomogram; green line: screen based on the PLT-MELD; blue line: screen based on the APRI.

In addition, we conducted DCA to confirm the clinical application value of the nomograms in predicting EGVs bleeding in patients with HBC. At a threshold probability of 6%–80%, application of the nomogram to predict EGVs bleeding in patients with HBC increased the benefit considerably compared to the PLT-MELD and APRI (Figure 5).

## Discussion

HBV infection remains the leading cause of chronic liver disease globally (1). In China, owing to the high infection rate of hepatitis B, there are a large number of patients with liver cirrhosis (21). EGVs are a common complication in patients with liver cirrhosis and the leading cause of death. However, the main factor that determines the bleeding of EGVs is portal hypertension (3). Splenomegaly is an early sign of portal hypertension. With the aggravation of portal hypertension, EGVs bleeding and hypersplenism will occur (4, 22). Previous studies have shown that portal vein diameter and liver/spleen volume ratio measured by computed tomography (CT) can predict the presence of portal hypertension and EGVs (10–12). Spleen volume, right liver volume, and liver volume correlated with the presence of EGVs (13–15). In this study, univariate and multivariate logistic regression analysis was performed on the imaging parameters and clinical laboratory test results of patients with HBC. It was determined that SVER, SNA, PT, and Hb were independent risk factors predicting EGVs bleeding in patients with HBC. A novel nomogram based on SVER for predicting EGVs bleeding was established effectively by combining CT imaging examination with clinical laboratory examination results.

The multivariate analysis results in this study showed that an increased SVER significantly increases the risk of EGVs bleeding in patients with HBC (OR = 1.20, 95% CI: 1.03–1.39, *P* = 0.020). SVER was positively correlated with EGVs bleeding in cirrhosis. In the progress of liver cirrhosis, the change of spleen volume indirectly reflects the pressure of the portal vein; the degree of portal hypertension is positively associated with the severity of EGVs. Increased pressure in the esophagogastric veins results that its vessel walls are thinning and becoming brittle, increasing the risk of bleeding. The results of this study are consistent with those of several studies. The findings of Sanjay, Amarapurkar et al. suggest that palpable splenomegaly is an independent predictor of large esophageal varices (10, 23). Yang et al. studied found that there was a statistically significant difference between the rate of spleen volume change and high-risk esophageal varices, and the rate of spleen volume change was negatively correlated with the degree of esophageal varices (8). For patients with liver cirrhosis, both palpable splenomegaly and the rate of change in spleen volume reflect that the size and volume of the spleen affect the progression of EGVs in liver cirrhosis. However, quantified SVER can more accurately and intuitively reflect the effects of spleen enlargement and spleen volume change ratio on esophagogastric varices in cirrhosis.

PT as a predictor of cirrhosis-related bleeding has been controversial. On the one hand, some studies suggest that PT cannot reliably predict the risk of bleeding in patients with liver cirrhosis (24). However, PT is abnormal in cirrhotic patients because it reflects a reduction in hepatic coagulation factor synthesis (25). Several studies have repeatedly pointed out that PT has never been shown to be a good predictor of bleeding in patients with cirrhosis undergoing liver biopsy or invasive surgery (26–28). It is important to note, however, that bleeding secondary to liver biopsy or invasive surgery can occur in anyone with independent liver disease (29). On the other hand, PT is a parameter that indicates the rate at which prothrombin is converted to thrombin, with low PT values indicating high conversion rates. With the exception of factor VIII, all other clotting factors are synthesized by the liver. Factors II, V, VII, and X are required for PT, and since these are made in the liver, the function of the liver is critical in the coagulation process (30). However, in patients with liver cirrhosis, due to the reduced synthesis function of the liver, the synthesis of various coagulation factors is reduced, and the PT is prolonged, which greatly increases the risk of bleeding in patients with liver cirrhosis, especially those patients at risk of bleeding from EGVs. The extent of PT prolongation depends on the level of factors synthesized by the liver and decreases as the synthesis capacity of the liver decreases (28). In addition, PT testing is inexpensive and readily available, and is generally considered to reflect the risk of bleeding. Therefore, PT is often used to predict bleeding risk in patients with hepatic insufficiency (28). Although PT is controversial as a coagulation test to predict bleeding in patients with cirrhosis, bleeding may be associated with changes in PT, at least according to our data analysis. Our multivariate analysis results show that PT was significantly associated with the occurrence of EGVs bleeding in patients with HBC (OR = 0.93, 95% CI: 0.87–1.00, *P* = 0.047). The study by Pilette et al. showed that the presence of esophageal varices can be correctly diagnosed in 71% of patients with cirrhosis by assessing PLT and PT alone, while the study by Schepis et al. also showed that PT is an independent predictor of EGVs in patients with cirrhosis (29, 31, 32). The results of this study are consistent with the above findings, suggesting that PT can be used as an indicator to predict the occurrence of EGVs bleeding in patients with liver cirrhosis. However, some authors argue that PT only measures the activity of a few procoagulant factors and cannot capture changes in other components necessary for hemostasis, as well as changes in anticoagulant activity that occur in liver disease. Changes in PT alone cannot reliably predict the bleeding risk (28, 29, 33, 34). Therefore, it is necessary to combine PT with other relevant predictors to improve the predictive value. In this study, we combined PT with other relevant independent risk factors and constructed a more reliable predictive model.

We found that SNA is an independent risk factor for the risk of bleeding from EGVs. To our knowledge, this has never been reported before. SNA was 139.8 (137.9, 142.0) mM and the MELD score was 14 (10, 17) in the group with EGVs bleeding; in the group without EGVs bleeding, SNA was 137.8 (134.3, 140.0) mM and the MELD score was 15 (12, 21). There was a statistical difference between the two groups (*P* < 0.05). These results show that SNA was negatively correlated with the MELD score in both groups. The previous study by Hou et al. also showed that the MELD score was negatively correlated with SNA (35). The multivariate analysis results showed that SNA was significantly associated with EGVs bleeding in patients with HBC (OR = 1.20, 95% CI: 1.01–1.24, *P* = 0.025). Levy et al. believe that there is a close relationship between sodium balance and blood volume. With the increase of portal venous pressure, the mesentery is hyperemic, and a large amount of blood accumulates in the open portosystemic collateral circulation. In a dog model of liver cirrhosis, it was demonstrated that sodium retention precedes the formation of ascites and any changes in systemic hemodynamics. Also, it has been shown that in the cirrhotic population, sodium retention can still be detected when the circulation is filled and the ascites disappear (36). However, in the collateral circulation caused by portal hypertension in cirrhosis, EGVs are present in more than 50% of patients (37). The results of these studies suggest that SNA plays a nonnegligible role in EGVs in patients with liver cirrhosis.

The overall mean Hb of patients with liver cirrhosis in this study was 101.51 ± 23.73 g/L, showing a state of mild to moderate anemia. In the group with EGVs bleeding, Hb was 95.29 ± 21.53 g/L, and it was 110.79 ± 24.00 g/L in the group without EGVs bleeding. Hb was significantly associated with EGVs bleeding in patients with HBC (OR = 0.98, 95% CI: 0.96–0.99, *P* = 0.006). In view of the results of this study, we believe that this may be because 85 (59.9%) of the patients included in this study had EGVs bleeding, which led to a lower Hb in the group with EGVs bleeding compared with the nonbleeding group. Most nonrenal erythropoietin (EPO) comes from the liver, and the normal liver can synthesize 10% of erythropoietin, but in patients with cirrhosis, owing to poor liver synthesis, decreased testosterone levels, the influence of pro-inflammatory feedback mechanisms, etc., the ability of the liver to synthesize EPO is decreased, which can lead to the occurrence of anemia (38, 39). In the development of cirrhosis, splenomegaly is an early sign of portal hypertension in cirrhosis. The splenic venous return resistance increases and the portal venous pressure is reversed to the spleen, resulting in passive congestive enlargement of the spleen and proliferation of spleen tissue and fibrous tissue. In addition, intestinal antigens enter the systemic circulation through the portal system collateral circulation and are absorbed by the spleen. The antigens stimulate the proliferation of spleen mononuclear macrophages and hypersplenism, which in turn leads to varying degrees of thrombocytopenia and leukopenia in peripheral blood and hyperplastic anemia.

The 2015 Baveno VI consensus emphasized that patients with liver stiffness measurements (LSM) < 20 kPa and platelet counts >150,000/mm^3^ are less likely to have high-risk varicose veins. Although this consensus has been validated by multiple studies (40–44). However, some scholars believe that the specificity of the Baveno VI criteria is relatively low, which increases the diagnosis of false-positive patients and the use of gastroscopes. During clinical practice, many patients have negative endoscopy results (45). Previous study has shown that platelet-MELD criteria can save more endoscopy compared to Baveno VI criteria. From a public health perspective, the platelet-MELD criteria can be promoted in medical settings where liver stiffness measurement cannot be widely performed (20). Therefore, we used the platelet-MELD criterion as a surrogate criterion for the Baveno VI consensus in our study. Compared with the Baveno VI standard, our model includes more and more comprehensive indicators. In this study, the prediction model we constructed included spleen volume, an index reflecting portal hypertension; Prothrombin time, hemoglobin concentration are indicators of liver synthetic function, and blood sodium concentration reflecting blood volume balance. The indicators included in the model can reflect the overall state of the patient in many ways. In addition, the models we build are easy to use, fast, and intuitive.

In general, male have an advantage in height and weight compared to female. Therefore, whether there are differences in spleen volume measured by CT in different genders. To address this question, we used logistic regression analysis to analyze changes in spleen volume between genders. Logistic regression analysis showed that there was no significant difference in spleen volume between genders (aOR = 1.001, 95%CI: 0.999–1.003, *P* = 0.151). In addition, we added gender to the model and calculated the area under the receiver operating characteristic curve (AUROC = 0.791, 95%CI: 0.714–0.868). Compared with the model constructed by us (AUROC = 0.781, 95%CI: 0.703–0.858), the AUROC area of the new model was larger. To comprehensively evaluate the gender of the two models, we evaluated the new model including gender using the Net Reclassification Index (NRI) and the Integrated Discriminant Improvement Index (IDI) (46, 47). The results showed: NRI (95% CI): −0.023 (95%CI: −0.172–0.082), *P* = 0.699; IDI (95% CI): −0.014 (95%CI: −0.034–0.005), *P* = 0.151. *P* values > 0.05 for NRI and IDI were not statistically significant, suggesting that the predictive power of the model did not improve in the new model with the addition of gender.

In this study, we show that it is feasible and clinically meaningful to construct a nomogram for predicting bleeding from EGVs in HBC patients based on the SVER. The AUROC of the nomogram was 0.781 (95% CI: 0.703–0.858), and cutoff value was 0.743, which corresponds to a sensitivity of 80.7% and a specificity of 63.5%. The calibration C-index was 0.757, and the calibration prediction curve fits well with the ideal curve. We found that SNA and Hb can be used as independent predictors of EGVs bleeding in HBC patients. This has not been proposed in previous studies. The major drawback of our study is that it is a single-center, small-sample study. Therefore, both multicenter and large-sample-size studies, as well as further prospective studies, are needed for validation to investigate whether the established nomograms have disease-specific cutoffs to identify the risk of bleeding from EGVs in HBC patients.

## Conclusion

In conclusion, the nomogram constructed based on the SVER can provide an excellent prediction of EGVs bleeding in HBC patients. External validation of the current model will be performed in future studies.

## Data Availability

The original contributions presented in the study are included in the article/Supplementary Material, further inquiries can be directed to the corresponding author/s.
